# Does Structural Violence by Institutions Enable Revictimization and Lead to Poorer Health Outcomes?—A Public Health Viewpoint

**DOI:** 10.5334/aogh.4137

**Published:** 2023-09-15

**Authors:** Gloria Macassa

**Affiliations:** 1 Department of Public Health and Sports Science, Faculty of Occupational and Health Sciences, University of Gävle, Kungsbacksvägen 47, 80176 Gävle, Sweden; 2 EPIUnit–Instituto de Saude Publica, Universidade do Porto, Rua das Taipas 135, 4050-600 Porto, Portugal; 3 Laboratório para a Investigação Integrativa e Translacional em Saúde Populacional (ITR), Rua das Taipas, nº135, 4050-600 Porto, Portugal; 4 Department of Public Health, School of Health Sciences, University of Skövde, 541 28 Skövde, Sweden

**Keywords:** institutional structural violence, interpersonal violence, revictimization, health, well-being

## Abstract

Although structural violence is known to interact with and reinforce direct violence in the form of interpersonal violence (e.g., intimate partner violence), little debate takes place in public health on how it can lead to revictimization, leading to even poorer health outcomes (including psychological ill health). This viewpoint aims to discuss this issue using examples from empirical studies to elucidate how structural violence (perpetrated through institutions) contributes to revictimization among people who are already suffering direct violence. Public health professionals (and researchers) need to make efforts to theorize and measure structural violence to aid efforts toward the study of how it intersects with interpersonal violence to influence health outcomes. This will ultimately contribute to better prevention and intervention efforts to curb interpersonal violence and improve population health and well-being. In addition, there is a need to include structural violence in the academic curriculum when training future generations of public health professionals. Increased education on structural violence will bring about an awareness of the grave consequences of the potential additional harm that institutions could inflict on the lives of people they should be protecting or care for.

## Background

This viewpoint departs from the concept of a triangle of violence put forward by Norwegian sociologist Johan Galtung [1]. He proposed that there are three types of violence that reinforce each other, arranged in a structure like that of an iceberg, in which there is a small visible part, though the largest part is hidden [1]. The tip of the iceberg, what is visible, is about direct (behavioral) violence, which in this viewpoint is understood as “interpersonal violence (IPV)”; this involves the intentional use of physical force or power against other persons by an individual or a small group of individuals [2]. Interpersonal violence may be physical, sexual or psychological (also called emotional violence), and it may involve deprivation and neglect [2]. Interpersonal violence can be further divided into family or partner violence and community violence [2,3].

At the base of the iceberg, there are two invisible types of violence: structural and cultural. “Structural violence (SV),” also called “indirect violence” and sometimes, “institutionalized violence,” refers to preventable harm or damage to persons (and by extension, things), where there is no single actor committing the violence or where it is not practical to search for the perpetrator(s) [1,4,5,6,7,8]. It is suggested that SV emerges from the unequal distribution of power and resources or is built into societal structures [1,4,5,6,7,8]. On the other hand, “cultural violence” is defined as “any aspect of a culture that can be used to legitimize violence in its direct or structural form [3].” Although SV is known to interact with and reinforce direct violence in the form of IPV, little debate takes place in public health on how it can perpetuate revictimization, which in turn, leads to even poorer health outcomes (e.g., psychological ill health).

To address this dearth of debate, this paper aims to discuss this issue using examples from empirical studies to elucidate how SV (perpetrated through institutions) contributes to revictimization among men and women who are already suffering direct violence. In this viewpoint, revictimization entails both victimization by institutions through SV (when victims do not get the appropriate help they need), as well through interpersonal violence (e.g., by partners or others) after the initial onset of IPV. However, these two revictimizations are intertwined and occur simultaneously.

First, through a chosen analysis of some empirical studies, this viewpoint identifies institutions and cultural norms that victimize female and male victims, causing further harm to their health and well-being; thereafter, it discusses the need for public health professionals, public health researchers and public health policy makers to be aware of and consider the invisible role of SV when developing IPV prevention strategies.

## Narratives of Structural Violence among Victimized Women and Men

While the existing research on the intersection between SV and IPV is scarce, there are some studies that have examined the revictimization of already victimized individuals. For instance, Flynn et al. reported that SV through exclusion and violence created a context that facilitated sexual violence revictimization and intimate partner violence [9]. Another study by Gillum in the USA found that women who experienced both poverty and IPV had very poor physical and psychological health [10]. In Spain, Sánchez-Sauco and colleagues reported that discrimination related to being a sex worker and an immigrant facilitated exposure to intimate partner violence and sexual harassment [11]. In Mexico, a mixed methods study revealed that victimized and incarcerated women returning to their rural communities were at an even greater risk of mental health and ill health, substance abuse and recidivism because of a compounding structural context—including criminalized interpersonal relationships and persistent racial and economic inequalities [12]. Women have also experienced institutional violence in situations where they needed help from social care institutions after victimization. For instance, in a study by Fleckinger [13], respondents felt that child protection social workers often adopted classic attitudes similar to those of perpetrators (e.g., intimidation, insisting on men’s rights and privileges, exploiting children, playing down and/or denying violence and/or blaming survivors) with the goal to maintain control over the victim [13].

Victimized men also suffer SV, especially at the hand of police, as well as in health care settings. Findings from a study by Dim and Lysova show that men who reported victimization by their female partners to the police were met with antagonistic and unfriendly attitudes; but worse was the reluctance of the police to charge their abusive partners [14]. Furthermore, there have also been accounts of structural revictimization, even in countries with a strong welfare state system (e.g., the Scandinavian countries) [15,16]. Pratt-Eriksson et al.’s study among abused women [15] found that their encounter with health care personnel was traumatic because of uncaring behaviors, such as a lack of support, care and empathy. In addition, a study of victimized women, some of whom had contact with social care services, found that they experienced loneliness—not just as a passive consequence of exposure to violence, but as a consequence of an actively lonely process at an interpersonal and a structural level [16]. According to the same report, this was due to the fact that societal responses to women’s exposure, resistance and attempts at emancipation often led to postviolence behavior from men, which left the women alone to face the violence and its consequences [16].

## Opportunities and Challenges of Including Structural Violence in Public Health Promotion and Interventions to Tackle Interpersonal Violence

As mentioned above, SV (as well as cultural violence) forms the base of the iceberg, which is much larger and invisible, and is therefore difficult to tackle. However, because this type of violence is insidious and ingrained in people’s everyday lives, it needs to be identified, as it can contribute to much poorer health outcomes among already victimized men and women. For instance, Gupta argues that unlike the more visible direct violence that can cause injury, SV occurs through economic, political and culture-driven processes that reinforce each other to limit victims from achieving full quality of life [17]. Montesanti and Thurston state that SV, symbolic violence and IPV are not mutually exclusive; rather, in their study, they were related to one another, as they manifested in the lives of the women they studied [18]. In the same study, SV was described as having unequal access to health determinants, such as employment, good quality of care and housing, which created conditions where IPV shaped the gendered forms of violence for women in the lowest social positions [18]. Moreover, some argue that at first glance, SV can be perceived as a misnomer for inequity and injustices that are characteristics of very stable social structures, where there is little overt disruption. In reality, however, SV is persistent and causes insidious and damaging harm [17].

The empirical evidence from narratives of women and men across the different contexts mentioned above illustrates how SV perpetrated through institutions and norms can cause injury, poor health and poor well-being. Winter and Leighton state that SV is human violence because the decisions are made by humans and are not a “natural occurrence”; moreover, SV can also be prevented through human intervention [19]. Also, it is important to understand that SV is invisible, subtle, accepted as a matter of course and difficult to detect; as a result, it is also difficult to assign culpability, and it is often not possible to identify SV’s perpetrators (as they are hidden behind anonymous institutions). So, it continues [1,5]. In addition, in dealing with invisible violence like SV, there are no concrete perpetrators directly attacking others, as compared to murder, for example [1,5]. Butchart and Engström [20] point to the fact that SV is by far the most lethal form of violence, as well as the most potent cause of other forms of violence, and the magnitude of the damage it causes warrants naming it “violence,” instead of “social injustice” and “oppression [20].”

From a public health perspective, most interventions aimed at curbing violence against women and men have failed to address the entirety of “Galtung’s triangle of violence.” Mostly, they have not addressed the base of the iceberg, where SV and cultural violence are located. This translates to victims being exposed to even greater suffering after the first onset of IPV has occurred.

In discussing this issue, St. Cyr and colleagues argued that there is a need for policies aiming to redress social and health inequities associated with partner violence through institutional and social change [12]. This viewpoint agrees with the assumption that only treating female and male violent victimization as “crime” tends to diminish the contribution of social and political institutions to IPV. Websdale and Johnson stated that it is unlikely that criminal justice would reduce the revictimization of already victimized women if there are no systematic policies in place to combat these women’s social and political disadvantages [21]. According to Montesanti and Thurston, public health responses to IPV need to look at how SV, IPV and power relations shape the lived experiences of violence for women and men alike [18].

It is noteworthy that several public health and health science researchers in the United States, Canada and Latin America have attempted to study the role SV plays in the health outcomes of directly victimized (or revictimized) individuals. This is concerning, since across the globe, IPV (as exposure) is less studied from a social inequality perspective (with the determinants of health framework), let alone through the lens of structural and cultural violence. It is argued that the social determinants of health and SV approaches have similarities, as they center around the unequal distribution of power—through social, economic and political systems—and generalized injustices that have a greater impact on people’s capacity to live healthy lives [8,22,23,24]. This absence of an SV lens for studying various outcomes, such as violence and health, is felt even more in Europe. For instance, a review of studies investigating how SV impacts health outcomes across Europe without time limits found only eight peer-reviewed studies that sought such a relationship [24].

Hyman and colleagues [25], analyzing data from a forum organized by the Centre for Global Health and Health Equity in 2014, proposed four recommendations that, in my view, are still valid today, especially as the intersection between IPV and SV across all genders is growing. First, they suggested that it is important to support and adopt policies that prevent or reduce SV; second, they point out that it is necessary to adopt multipronged strategies to transform dominant social norms associated with violence; third, they propose establishing appropriate standards and ensuring adequate and sustained funding for violence prevention programs and services; and fourth, they emphasize the importance of carrying out ecological-level research (which incorporates the structural systems and social norms) on violence prevention and mitigation [25].

With regards to interventions, considering also the role played by SV on IPV, a systematic review of the impact of structural interventions for male to female IPV in low- and middle-income countries found that 13 of the 16 reviewed studies showed statistically significant effects for at least one primary or secondary outcomes [26]. The outcomes included decreased IPV and controlling behaviors; enhanced relationship quality; improvements in empowerment, economic well-being and social capital; reduced acceptability of IPV; more equitable gender norms; and new help-seeking behaviors [26]. However, in general, and as already mentioned above, there is a dearth of IPV interventions tackling SV. One explanation may be due to the complexity of measuring SV. De Maio and Ansell suggest that SV needs to be seen as a complex concept with rich explanatory potential but, at the same time, is vague in its operationalization and lack of theoretical precision [8]. They further posit that, contrary to the notion of social determinants of health, which is a central pillar of social epidemiology, SV focuses on the roots of health inequalities that go much deeper, as SV attempts to identify social, economic and political systems as the causes of poor health outcomes [8]. The subject of the intersectionality of SV and IPV is less studied in the field of public health sciences, let alone being included in the educational curriculum of public health scientists. In addition, De Maio and Ansell argue that contrary to the most theorized notion of social determinants, which social epidemiology (a subdiscipline of public health) relies on, SV focused on the root causes of health inequalities, which went further than individual-level constructs (e.g., education, occupation and income) to identify the social, economic and political systems as causes of poor health outcomes [8].

In their seminal paper mapping the role of structural and interpersonal violence in the lives of women and their implications for public health interventions and policy, Montesanti and Thurston stated that the accounts of the daily lived experiences of abused or assaulted women highlighted the intersections of micro, meso and macro levels in the production and reproduction of violence. Furthermore, they argued that the results of the mapping showed women experienced interpersonal violence not only in direct physical harm but also through injury caused by the bureaucracies within institutions that did not respond to their needs and instead disrespected and mistreated them—further exacerbating their marginalization [18]. I argue that similar situations, as demonstrated by Montessanti and Thurston, are likely to occur among male victims of IPV.

Public health research has had an opportunity to add the concept of SV when addressing inequalities in health. This has to some degree been a result of researchers from other disciplines (e.g., anthropologists and sociologists) attempting to understand how SV associates with health-related outcomes [24]. However, a central challenge still exists regarding theorization, as well as the measurement of SV and its impact on IPV, as well as health outcomes. In a recent paper on the evolutionary concept analysis of structural violence, Jackson and Saddler point out that the lack of conceptual clarity and operationalization of SV prevents the identification of measurable constructs of SV [27]. Moreover, they indicated the need for awareness of the concept of SV and its interdisciplinary usage that could validate and help hypothesis-building in relation to health and well-being [27]. In this viewpoint, I agree with Raguz’s suggestion—that violence is multifaceted (physical, psychological, sexual, personal, social, institutional, cultural and historical) and that there is a need to address it using a comprehensive approach, one that takes into consideration the intersectional approach (which can include interpersonal as well as structural factors) [28].

## Conclusion

This viewpoint calls for public health professionals to be aware of the role structural and cultural violence plays in the revictimization and worsening health and well-being of victims of IPV. This type of violence can take a variety of forms, which are hard to detect as they are institutionalized (e.g., health and social and care institutions, police and criminal justice) and invisible to anyone, and therefore go unchallenged. Thus, it is important that public health researchers continue to make efforts to theorize and measure SV (and CV), as well as understand how it connects with the notion of social determinants, which is already widely used within the discipline (more specifically within social epidemiology). A better understanding of SV and how it intersects with IPV will have an impact on future interventions aimed at minimizing revictimization and improving the health outcomes among victimized women and men. Beyond that, there is a need for structural and cultural violence to become part of the academic curriculum when training future generations of public health, health care and social care professionals. Increased education on SV can raise awareness of the grave consequences of the potentially added harm institutions might inflict on the lives of people they are expected to protect or care for in the first place.
